# Circumstances for treatment and control of invasive Enterobacterales infections in eight hospitals across sub-Saharan Africa: a cross-sectional study

**DOI:** 10.12688/gatesopenres.14267.1

**Published:** 2023-02-01

**Authors:** Alexander M. Aiken, Brian Nyamwaya, Lola Madrid, Dumessa Edessa, Appiah-Korang Labi, Noah Obeng-Nkrumah, William Mwabaya, Mabvuto Chimenya, Derek Cocker, Kenneth C. Iregbu, Philip I. P. Princewill-Nwajiobi, Angela Dramowski, Tolbert Sonda, Blandina Theophil Mmbaga, David Ojok, Sombo Fwoloshi, J Anthony G Scott, Andrew Whitelaw, MBIRA study collaborators

**Affiliations:** 1 Infectious Disease Epidemiology Department, London School of Hygiene and Tropical Medicine, London, UK; 2 KEMRI Centre for Geographic Medicine Research, Kilifi, Kenya; 3 School of Pharmacy, Haramaya University, Harar, Ethiopia; 4 Department of Medical Microbiology, University of Ghana Medical School, Accra, Ghana; 5 Department of Medical Laboratory Sciences, University of Ghana, Accra, Ghana; 6 Malawi-Liverpool Wellcome Programme, Kamuzu University of Health Sciences, Blantyre, Malawi; 7 Department of Clinical Sciences, Liverpool School of Tropical Medicine, Liverpool, UK; 8 Department of Medical Microbiology, National Hospital Abuja, Abuja, Nigeria; 9 Department of Paediatrics and Child Health, Faculty of Medicine and Health Sciences, Stellenbosch University, Cape Town, South Africa; 10 Kilimanjaro Clinical Research Institute-Kilimanjaro Christian Medical Centre, Moshi, Tanzania; 11 Department of Paediatric and Child Health, Kilimanjaro Christian Medical University College, Moshi, Tanzania; 12 Centre for Infectious Disease Research in Zambia, Lusaka, Zambia; 13 Department of Medicine, University Teaching Hospital, Ministry of Health, Lusaka, Zambia; 14 National Health Laboratory Service, Tygerberg Hospital, Cape Town, South Africa; 15 Division of Medical Microbiology, Faculty of Medicine and Health Sciences, Stellenbosch University, Cape Town, South Africa

**Keywords:** antibiotic, access, infection, resistance, Africa

## Abstract

**Background:** Bloodstream infections caused by Enterobacterales show high frequency of antimicrobial resistance (AMR) in many Low- and Middle-Income Countries. We aimed to describe the variation in circumstances for management of such resistant infections in a group of African public-sector hospitals participating in a major research study.

**Methods:** We gathered data from eight hospitals across sub-Saharan Africa to describe hospital services, infection prevention and antibiotic stewardship activities, using two WHO-generated tools. We collected monthly cross-sectional data on availability of antibiotics in the hospital pharmacies for bloodstream infections caused by Enterobacterales. We compared the availability of these antibiotics to actual patient-level use of antibiotics in confirmed Enterobacterales bloodstream infections (BSI).

**Results:** Hospital circumstances for institutional management of resistant BSI varied markedly. This included self-evaluated infection prevention level (WHO-IPCAF score: median 428, range 155 to 687.5) and antibiotic stewardship activities (WHO stewardship toolkit questions: median 14.5, range 2 to 23). These results did not correlate with national income levels. Across all sites, ceftriaxone and ciprofloxacin were the most consistently available antibiotic agents, followed by amoxicillin, co-amoxiclav, gentamicin and co-trimoxazole. There was substantial variation in the availability of some antibiotics, especially carbapenems, amikacin and piperacillin-tazobactam with degree of access linked to national income level. Investigators described out-of-pocket payments for access to additional antibiotics at 7/8 sites. The in-pharmacy availability of antibiotics correlated well with actual use of antibiotics for treating BSI patients.

**Conclusions:** There was wide variation between these African hospitals for a range of important circumstances relating to treatment and control of severe bacterial infections, though these did not all correspond to national income level. For most antibiotics, patient-level use reflected in-hospital drug availability, suggesting external antibiotics supply was infrequent. Antimicrobial resistant bacterial infections could plausibly show different clinical impacts across sub-Saharan Africa due to this contextual variation.

## Background

Antimicrobial resistance (AMR) is a global challenge with major implications for African countries where severe bacterial infections are common, but access to antibiotics is often limited ^1, 2^. Recent estimates suggest that the population mortality rate linked to bacterial AMR is highest in sub-Saharan Africa ^3^. Bloodstream infections (BSI) caused by Gram-negative pathogens including Enterobacterales are important contributors to neonatal, child and adult morbidity and mortality in Africa ^3– 6^. High prevalence of 3 ^rd^ generation cephalosporin (3GC) resistance amongst *E. coli* and *K. pneumoniae* causing BSI has been reported from many African countries, often substantially exceeding proportions found in high-income settings ^3, 7– 10^. Prevalence of resistance to many other antibiotic agents in Enterobacterales in Africa is also thought to be concerningly high.

Many African hospitals lack local microbiological testing services and have relatively limited resources for preventative activities to minimize the impacts of bacterial AMR, including infection prevention and control (IPC) and antimicrobial stewardship. Furthermore, in many African hospitals, the availability of “watch” and “reserve” antibiotic agents ^11^ for treating resistant infections is limited, though precise data describing day-by-day supply of particular agents are scarce ^12^. We are not aware of any previous comparisons between the availability of antibiotics with actual patient-level usage in hospital settings in Africa.

As part of a multi-centre cohort study across African hospitals designed to measure mortality impact effects associated with bacterial AMR (the MBIRA study), we aimed to describe the baseline circumstances in the participating hospitals in terms of their capacity to treat patients with resistant bacterial bloodstream infections caused by Enterobacterales.

## Methods

**Study setting:** Eight public hospitals from eight different sub-Saharan African countries were invited to participate. These were Tygerberg Hospital, Cape Town, South Africa; Korle-Bu Teaching Hospital, Accra, Ghana; National Hospital, Abuja, Nigeria; Kilifi County Hospital, Kilifi, Kenya; University Teaching Hospital, Lusaka, Zambia; Kilimanjaro Christian Medical Centre, Moshi, Tanzania; Hiwot Fana Comprehensive Specialized Hospital, Harar, Ethiopia; Queen Elizabeth Central Hospital, Blantyre, Malawi. These hospitals were purposively selected, primarily on the basis of having well-established microbiology services including performance of blood cultures, with relevant indicators of laboratory quality control measures. Preliminary discussions were held with several other hospitals, but they were not formally invited to participate as they did not meet the inclusion criteria.

**Data collection**: at the start of the MBIRA study, each site gathered data at the hospital level. This included data on the number of general and intensive care beds in the hospital, assessing the level of intensive care beds with reference to a common international standard ^13^. We categorized intensive care units from level 1 (lowest) to level 3 (highest) using objective criteria, including therapeutic capacity, personnel and monitoring capacity. Laboratories reported historic blood culture activity levels for 2019 (where available) and prospectively reported total monthly blood cultures performed during the period of active patient recruitment in 2020–22. Laboratories self-assessed which blood culture isolates represented contamination, using standardized recommendations ^14^. 

As part of this assessment, we used two externally created tools: the “WHO Infection Prevention and Control Assessment Framework at Facility Level” (hereafter “WHO-IPCAF”) ^15^ and the “Antimicrobial Stewardship programmes in health-care facilities in low and middle-income countries – a WHO practical toolkit” (hereafter “WHO stewardship toolkit”) ^16^. The WHO-IPCAF questionnaire self-assesses facility-level IPC activity via questions in eight core components ( Table 2), each scored out of 100 for a maximum possible total of 800. According to the interpretation provided, a score of <200 represents an “inadequate” level of IPC, 200–400 represents a “basic” IPC level, 400–600 represents “intermediate” IPC level and >600 represents “advanced” IPC level. This WHO-IPCAF tool has previously been shown to have adequate internal consistency and good inter-observer reliability ^17^. In practice, one large hospital in Ghana repeated the WHO-ICPAF survey with seven specialists from different departments across the facility; we used the arithmetic mean of the scores obtained as a facility-wide score. All other hospitals used a single scoring with local investigators identifying relevant hospital staff for different questionnaire elements.

The WHO stewardship toolkit includes a series of 31 yes/no questions grouped into six domains ( Table 2); these questions are designed for self-assessment of current implementation of antimicrobial stewardship activities at the facility level. Local investigators identified suitable hospital staff for response to different questions, who provided informed oral consent to participate. We summarized these questions by awarding 1 point for each “yes” response. We are not aware of any published validation studies of this tool, which appears to have been primarily designed for within-facility quality-improvement exercises. Both the WHO-IPCAF and the stewardship tools contain extensive descriptions of the intended approaches to data collection. 

During the active period of patient recruitment in the main study, each MBIRA hospital team completed monthly cross-sectional pharmacy surveys on the availability of antibiotic agents suitable for the treatment of bloodstream infections caused by Enterobacterales. The survey was completed on a working weekday at a regular timepoint in each month, with a local pharmacist and a study investigator jointly assessing antibiotic availability by examining the drug stock physically held in the pharmacy at that time. Where a large hospital had more than one pharmacy, the current antibiotic availability in the main or central pharmacy was surveyed. We considered an antibiotic as “available” when any quantity of the drug of any formulation was found to be “in stock”, provided that it was within the manufacturer’s expiry date. We considered an antibiotic as “not available” in a particular month when the drug was reported as “not normally available” or “out of stock” on the particular day of the survey. We identified 11 antibiotics drugs that are all included in the WHO Essential Medicines List Antibiotic Book formulary (either under “Access” or “Reserve” classes of AWARE classification) to gather individual drug availability data. We summarized the results of these repeated surveys by drug and by hospital by taking an arithmetic average of these binary results across all relevant surveys, expressing the result as a percentage. Each hospital also provided a written response to a standardized question about the particular approaches or special circumstances that applied (in the experience of the most senior local clinical investigator) for gaining access to antibiotic drugs for treatment of antibiotic resistant infections, if suitable agents were not immediately available through routine supplies.

For patient-level antibiotic use information, hospital inpatients of all ages with culture-confirmed bloodstream infections (BSI) caused by Enterobacterales bacteria (including *E. coli*, *K. pneumoniae* and various other species, but excluding *Salmonella spp.*) were prospectively recruited as part of the main work of the MBIRA study. These patients (or their relatives for children) provided written informed consent to participate in the study and were followed up whilst inpatients, including recording of day-by-day antibiotic use up to 30 days after BSI onset. Full description of these BSI patients and exploration of the impacts of AMR is planned for a subsequent publication. Here, we plotted the proportion of study months in which these antibiotic drugs were available in the hospital pharmacies (x-axis) against the collapsed proportions of *Enterobacterales* BSI patients at each hospital over the same period who received at least one drug-day of treatment with the respective drug (y-axis). Data collection on antibiotic use for BSI patients was limited from the day prior to blood culture collection to the cessation of antibiotic treatment or 30 days after blood culture collection, whichever was sooner.

Contextual information on national-level income categories and Gross Domestic Product (GDP) per head were obtained from the World Bank using 2020 data (Purchasing Power Parity, current international $ ^18^) . Hospital-level and pharmacy-level data were entered into an online REDcap database. Statistical tests were performed using STATA (v16, Statacorp, USA).

**Study approvals:** Ethical approval was granted by the relevant health research ethics committee at each site as follows:

- London School of Hygiene and Tropical Medicine on 9/7/2020 (21236)
- KEMRI Scientific and Ethics Review Unit on 2/12/2020 (KEMRI/SERU/CGMR-C/CSC/205/4105)
- National Commission for Science, Technology and Innovation (NACOSTI) on 26/11/2020 (BAHAMAS ABS/P/20/7869)
- Korle Bu Teaching Hospital on 18/11/020 (KBTH-IRB/00097/2020)
- National Health Research Authority Zambia on 17/2/2021
- University of Malawi College of Medicine Research and Ethics Committee on 2/11/2020
- Ministry of Science and Higher Education, Ethiopia on 24/2/2021 (MOSHE\1-16\10.81\13)
- Health Research Committee, National Hospital Abuja on 8/7/2020 (NHA/EC/041/2020)
- Tygerberg Hospital (N20/02/072)
- National Institute for Medical Research, Tanzania on 11/12/2020 (NIMR/HQ/R.Sa/Vol.IX/3575)

Permissions to access the laboratory and hospital registers were obtained from the relevant authorities in each hospital.

## Results

The eight hospitals participating in the MBIRA study collected the hospital-level data between October 2020 and March 2021. Pharmacy-level data was collected monthly in each hospital for between 8 and 15 months between November 2020 and February 2022, corresponding to the months of active participant recruitment to the main MBIRA study. Antibiotic use data was obtained from 996 patients with confirmed Enterobacterales bloodstream infection in these hospitals.

**Hospital-level characteristics ( Table 1).** These hospitals were all public-sector tertiary-level hospitals with some prior experience of clinical research. Seven of the eight hospitals were based in major urban areas – either in a city or the national capital. The eight hospitals were distributed across countries which reflect the substantial spread of income-levels across sub-Saharan Africa, ranging from the “upper-middle income” (South Africa) to “low income” (Ethiopia and Malawi). There was substantial variation in the number of intensive care beds available in relation to the size of the hospital. This ranged from no beds meeting an enhanced care definition available at all in a hospital in Kenya, to a modest number of Level 1 and Level 2 beds in hospitals in Tanzania, Ethiopia and Malawi, to more substantial numbers of Level 3 beds in hospitals in Nigeria and South Africa. In terms of blood culturing practices, in 2019 these hospitals all reported performing more than 100 blood cultures/1,000 patient admissions, where suitable data was available to calculate this metric. In 2021, the hospitals in Malawi and South Africa performed the largest absolute number of blood cultures, both averaging over 1,000 cultures per month. One hospital in Nigeria performed a relatively low number of blood cultures in 2021 (average of 90/month) – this was partly due to two prolonged national medical strikes in Nigeria that year. All other participating hospitals performed an average of more than 100 blood cultures per month in the MBIRA study period. Blood culture contamination rates varied widely, from 3.6% to 25%. 

**Table 1.** Participating institution descriptions – national, hospital and laboratory characteristics.

	Hospital 1, South Africa	Hospital 2, Ghana	Hospital 3, Nigeria	Hospital 4, Kenya	Hospital 5, Zambia	Hospital 6, Tanzania	Hospital 7, Ethiopia	Hospital 8, Malawi
Income category level, 2020, World Bank ^18^	Upper-middle	Lower-middle	Lower-middle	Lower-middle	Lower-middle	Low-middle	Low	Low
GDP / capita PPP, 2020, World Bank (current international US$) ^18^	13,518	5,552	5,132	4,475	3,358	2,691	2,377	1,571
Hospital location	City	Capital city	Capital city	Town	Capital city	City	City	City
Number of beds	1,342	1,538	425	200	846	721	238	1,350
Highest level ICU beds (n)	Level 3 (50)	Level 3 (11)	Level 3 (20)	None	Level 2 (18)	Level 2 (15)	Level 2 (10)	Level 3 (4)
Highest level PICU beds (n)	Level 3 (10)	Level 2 (42)	None	None	Level 2 (8)	Level 1 (5)	Level 1 (6)	Level 3 (6)
Highest level NICU cots (n)	Level 3 (12)	Level 2 (63)	Level 3 (18)	None	Level 2 (90)	Level 2 (3)	Level 1 (41)	Level 2 (50)
Admissions/ year (2019)	106,451	49,648	9,215	7,552	19,947	18,892	13,530	Data not available
BC in 12 month period (2019)	16,363	Data not available	1,190	4,513	Approx. 5,000	2,786	2,856	22,383
BC/1,000 admissions (2019)	154	N/A	129	598	Approx. 250	147	211	N/A
Mean monthly BC performed during study period (2020–22)	1,351	430	90	304	259	269	186	1,056
Mean BC contamination % during study period (2020–22)	7.0%	15.0%	15.4%	3.6%	12.2%	10.5%	25.2%	13.1%
External support for BC in 2021	Nil	FF + Research	FF + Research	Research	FF	FF	Research	Research
External laboratory accreditation (as of October 2020)	Yes (SANAS)	In process (CDC/WAPHL)	No	Yes (Qualogy)	No	Yes (SADCAS, Qualogy)	No	In process (SADCAS)

Abbreviations used: GDP = Gross Domestic Product, PPP = purchasing power parity, ICU/PICU/NICU = (Paediatric/Neonatal) Intensive Care Unit, BC = blood cultures, FF = Fleming Fund, SANAS = South African National Accreditation System, CDC/WAPHL = Centers for Disease Control and Prevention/West African Association of Public Health Laboratories, SADCAS = Southern African Development Community Accreditation Services.

**WHO-IPCAF toolkit ( Table 2)**. All hospitals self-completed the WHO-IPCAF questionnaire. The median total WHO-ICPAF score for these facilities was 428/800 with a range of 155 to 687.5 ( Table 2). Across all the hospitals, the two lowest-scoring IPC core components were “Hospital-acquired infection surveillance” (mean score 34/100) and “Workload, staffing and bed occupancy” (mean score 37/100). We found no evidence of a relationship between World Bank national income level categories and the hospital-level WHO-IPCAF scores (Kruskall-Wallis equality-of-populations rank test, p=0.83).

**Table 2.** Profile of self-reported Infection Prevention and Control and Antibiotic Stewardship practices.

Parameter	Hospital 1, South Africa	Hospital 2, Ghana *	Hospital 3, Nigeria	Hospital 4, Kenya	Hospital 5, Zambia	Hospital 6, Tanzania	Hospital 7, Ethiopia	Hospital 8, Malawi
**WHO- IPCAF score**								
CC1. IPC programme (/100)	77.5	36.7	70	72.5	35	92.5	72.5	10
CC2. IPC guidelines (/100)	52.5	44.3	87.5	65	52.5	100	80	37.5
CC3. IPC education+training (/100)	45	45.7	80	75	35	80	95	20
CC4. HAI surveillance (/100)	40	22.8	47.5	7.5	37.5	95	10	15
CC5. Multimodal strategies for IPC intervention implementation (/100)	55	38.7	55	75	35	100	90	0
CC6. Monitoring of IPC practices (/100)	57.5	45.5	65	77.5	45	90	67.5	5
CC7. Workload, staffing, bed occupancy (/100)	5	27.9	70	15	65	30	55	30
CC8. Environment, materials and equipment for IPC (/100)	70	44.1	67.5	65	72.5	100	72.5	37.5
**Total (/800)**	**402.5**	**305.7**	**542.5**	**452.5**	**377.5**	**687.5**	**542.5**	**155**
Corresponding IPC level	Intermediate	Basic	Intermediate	Intermediate	Basic	Advanced	Intermediate	Inadequate
**WHO Stewardship toolkit score (number of “yes” responses)**								
1. Leadership commitment (/3)	0	0	1	0	2	2	2	0
2. Accountability and responsibilities (/7)	1	0	6	2	4	7	6	0
3. AMS actions (/10)	7	2	1	7	4	7	4	5
4. Education and Training (/3)	0	0	1	3	1	3	0	1
5. Monitoring and surveillance (/4)	1	0	3	4	2	3	3	2
6. Reporting and feedback (/4)	0	0	3	2	1	1	2	1
**Total (/31)**	**9**	**2**	**15**	**18**	**14**	**23**	**17**	**9**

* IPCAF survey performed with 7 different individuals in Hospital 2 in Ghana as large hospital complex, mean of scores used.

**WHO stewardship toolkit ( Table 2)**. All hospitals completed responses for the 31 yes/no questions in the WHO stewardship toolkit. The range of self-assessed total scores varied very widely (2 to 23), with a median of 14.5 “yes” responses. Amongst the different categories, the “Leadership commitment” and “Reporting and Feedback” questions were least likely to have “yes” responses and the “Monitoring and feedback” questions were most likely to have “yes” responses. We found no evidence of an association between national income level and facility WHO stewardship toolkit overall score (Kruskall-Wallis equality-of-populations rank test, p=0.64). However, there was strong evidence of association between the hospitals’ scores for the WHO-IPCAF questionnaire and the WHO stewardship toolkit (Spearman’s rank correlation coefficient, p<0.01). 

**Cross-sectional antibiotic availability surveys ( Table 3)**. In terms of local availability of antibiotic drugs suitable for treatment of invasive Gram-negative infections, all the hospitals completed at least 8 monthly cross-sectional surveys (range 8–15) in their main hospital pharmacies. We found a marked variation in the availability of these antibiotic agents across the eight hospitals - see Table 3. In one hospital in South Africa (the only upper-middle income country in the study), all of these 11 agents were available in all rounds of the cross-sectional survey. In all of the other hospitals, there were one or more antibiotic agents that were only intermittently available, meaning that the drug was recorded as “not available” in at least one monthly cross-sectional survey. This phenomenon was especially marked in the study hospitals in Malawi (8 agents with intermittent availability) and Zambia (7 agents with intermittent availability). Finally, there were various antibiotic drugs that were “never available”, meaning that none of the cross-sectional surveys reported availability of that particular agent in that hospital. This occurred most commonly in the study hospitals in Ethiopia (3 agents never available) and Tanzania (2 agents never available).

**Table 3.** Monthly surveys of hospital pharmacy availability of antibiotic agents for treating Gram negative infections.

	AWARE category	Hospital 1, South Africa	Hospital 2, Ghana	Hospital 3, Nigeria	Hospital 4, Kenya	Hospital 5, Zambia	Hospital 6, Tanzania	Hospital 7, Ethiopia	Hospital 8, Malawi	Availability across all sites, by agent
N of rounds of survey		8	12	15	12	10	10	9	13	89
Amoxicillin	Access	8/8	9/12	13/15	12/12	2/10	10/10	9/9	12/13	75/89, 84%
Co-amoxiclav	Access	8/8	12/12	15/15	12/12	1/10	7/10	9/9	4/13	68/89, 76%
Piperacillin-tazobactam	Watch	8/8	3/12	9/15	0/12	6/10	3/10	0/9	7/13	36/89, 40%
Ceftriaxone	Watch	8/8	12/12	15/15	12/12	10/10	10/10	8/9	11/13	86/89, 97%
Cefotaxime	Watch	8/8	6/12	3/15	0/12	10/10	0/10	9/9	1/13	37/89, 42%
Meropenem	Watch	8/8	12/12	14/15	4/12	6/10	1/10	0/9	11/13	56/89, 63%
Gentamicin	Access	8/8	11/12	15/15	11/12	2/10	7/10	9/9	13/13	76/89, 85%
Amikacin	Access	8/8	11/12	15/15	4/12	6/10	0/10	0/9	1/13	45/89, 51%
Ciprofloxacin	Watch	8/8	12/12	15/15	11/12	10/10	10/10	9/9	13/13	88/89, 99%
Chloramphenicol	Access	8/8	0/12	15/15	1/12	4/10	2/10	1/9	8/13	39/89, 44%
Co-trimoxazole	Access	8/8	5/12	14/15	12/12	10/10	8/10	9/9	13/13	79/89, 89%
Availability of all agents, by site		88/88 100%	93/132 70%	143/165 87%	79/132 60%	67/110 61%	58/110 53%	63/99 64%	94/143 66%	
Other agents typically available for resistant Gram-negative infections		Cefepime, ertapenem, imipenem, ceftazidime, colistin	Ceftazidime	Imipenem	Ceftazidime	Ceftazidime, ceftriaxone- sulbactam		Ceftazidime		

We found that the antibiotics ceftriaxone and ciprofloxacin were the most consistently available antibiotic agents across these eight hospitals – these were available in almost all cross-sectional surveys in all these hospitals (both >95% availability across all survey rounds). The antibiotics amoxicillin, co-amoxiclav, gentamicin and co-trimoxazole were all found to be widely, but not universally, available (all available 70% to 90% across all survey rounds). The antibiotics piperacillin-tazobactam, meropenem, amikacin, cefotaxime and chloramphenicol were only partially available across these hospitals (all available 40% to 65% across all survey rounds). Few hospitals (2/8) described any availability of other β-lactam-β-lactamase-inhibitor agents or colistin for treating resistant Gram-negative infections.

 All hospitals provided a description of the local special circumstances for accessing antibiotics for treating resistant infections – Table 4. We identified three common themes in these responses: 1) patients buying antibiotics from external private pharmacies using either out-of-pocket payments or personal/private insurance (mentioned at 7/8 hospitals); 2) additional provision of antibiotic access through national health insurance (mentioned at 3/8 hospitals); 3) other access routes using external funding such as charitable donations or research-associated supply (mentioned at 2/8 hospitals). Only the South African hospital described rarely experiencing any antibiotic non-availability, whilst the hospital in Malawi emphasized that difficulties in antibiotic access were “commonplace”.

**Table 4.** Description of “special circumstances” for gaining access to antibiotic agents for treatment of antibiotic-resistant infections.

Location	Description provided by local lead investigator
**Hospital 1,** ** South Africa**	Owing to good supply chain management, our hospital seldom experiences stock outs of key antimicrobials unless there are national/international supply constraints from the manufacturing side.
**Hospital 2,** ** Ghana**	Patients on the national health insurance scheme access antibiotics in private accredited pharmacies. Rarely, a few patients can access antibiotics within and outside the hospital using private insurance. Patients without health insurance access antibiotics from the hospital pharmacies or private pharmacies and pay out of pocket.
**Hospital 3,** ** Nigeria**	Patients are given the prescription to source for the drugs from external pharmacy stores in town or elsewhere. For high class patients who pay between two to three times the normal cost, the pharmacy undertakes the sourcing of the out of stock drugs for them.
**Hospital 4,** ** Kenya**	Drugs are first sourced from the [on-site research-associated] Pharmacy’ using a prescription; if unavailable, patients are sent to purchase from private pharmacies.
**Hospital 5,** ** Zambia**	We write prescription the patient purchases from private pharmacy. If they have national health insurance that's they can collect from private pharmacy that is approved by our national insurance. The hospital does buy, we rarely use this route as it's lengthy.
**Hospital 6,** ** Tanzania**	If the patient is insured by the National Health Insurance Company (NHIC), a special form known as form C is usually written that refers the patient to get the med from other private pharmacies authorized by the NHIC without out of pocket payment. Two, if the patient is not insured, doctors usually tend to shift to another med with similar efficacy and available at the hospital pharmacy or the patient is advised to buy the prescribed meds from private pharmacies.
**Hospital 7,** ** Ethiopia**	The only available option is to buy them in private pharmacies by showing medical prescriptions. Since some of the antibiotics prescribed in this approach are prohibitively unaffordable for most of the families of patients admitted to this public hospital, the option is not routinely used.
**Hospital 8, ** **Malawi**	It is commonplace for patients or their families to purchase their own medications in the event of pharmacy stockouts, when alternatives are not available. Hospital departments can utilize charitable funds to purchase medications that are then internally stocked, and occasionally medicines can be accessed from non-QECH pharmacies including local NGOs or subsidiary [Ministry of Health] bodies (Lighthouse for HIV) or via recruitment into research studies.

**Figure 1.**
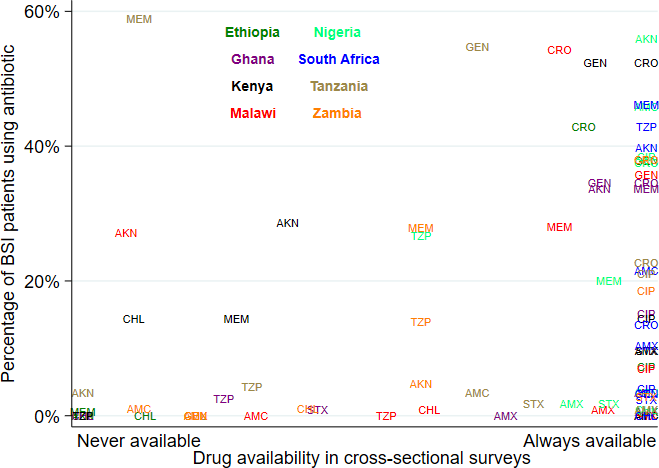
Pharmacy availability of antibiotic drugs versus documented use in Enterobacterales BSI patients. AMX = amoxicillin, AMC = co-amoxiclav, GEN = gentamicin, AKN = amikacin, CIP = ciprofloxacin, CRO = ceftriaxone, MEM = meropenem, CHL = chloramphenicol, STX = co=trimoxazole, TZP = piperacillin-tazobactam; figure does not include cefotaxime

In a scatter plot of the in-pharmacy availability of antibiotic drugs against percentage of local BSI patients with recorded use of each drug ( Figure 1), we found that for most antibiotics in most hospitals, there was a correlation between measured pharmacy availability and actual use for treating BSI patients. When specific antibiotics were infrequently available in hospital pharmacies, they were rarely used for treating patients with BSI. There were a small number of exceptions to this broad pattern, most notably the usage of meropenem in the hospital in Tanzania and of amikacin in the hospital Malawi, which were both commonly used despite rarely being available in the hospital pharmacy.

## Discussion

We found some variation in contextual circumstances for treatment and prevention of antibiotic resistant infections across eight public-sector hospitals in different African countries, across the range of national income levels in sub-Saharan Africa. Although these were all tertiary hospitals with some research orientation and mainly located in major cities, the levels of institutional capacity for dealing with antibiotic resistant infections varied markedly both at institutional level (IPC and antibiotic stewardship activities) and at individual-patient level (access to relevant “watch” and “reserve” antibiotics). We discuss both common themes and areas of variation. 

Using the WHO-IPCAF tool, we found a median self-assessed facility-level score of 428/800, which corresponds to an “intermediate” overall level of IPC compliance, according to the developers of this tool. There was a wide variation in the self-assessed score achieved from these eight hospitals (range 155 to 687.5), but there were common themes of poor performance in particular domains (‘Healthcare associated infection (HAI) surveillance’ and ‘Workload, staffing and bed occupancy’). We compared our findings to other studies that used the WHO-IPCAF tool. A recent major study described use of this WHO tool in over 4,400 hospitals worldwide ^19^. In that study, the weighted median (IQR) IPCAF score for 595 hospitals in Africa was 415/800 (290-582), with “HAI surveillance” also identified as an area of notable weakness (median score 32/100). These are extremely similar findings to our own results, suggesting that in terms of IPC activities, our 8 hospitals are broadly similar to hospitals across the continent. In terms of other published reports, a study of five hospitals in Pakistan reported a median WHO-IPCAF score of 117.5/800, with all hospitals scoring <200/800, representing substantially worse IPC scoring than we found. The authors attributed these low scores to non-existent IPC guidelines and HAI surveillance ^20^. A large study from Germany ^21^ included more than 700 hospitals and reported a median WHO-IPCAF score in the “advanced” level (677/800) of IPC compliance.

The low WHO-IPCAF scores found in our study might be partly explained by the resource constraints experienced in most public hospitals in Low- and Middle-Income Countries (LMIC), as others have found ^19^. However, we did not find an association between the WHO-IPCAF scores and national-level income data; the highest WHO-IPCAF score was from a hospital in Tanzania, whereas the only hospital from an “upper-middle income” nation (South Africa) had a score close to our median value. This suggests it is not purely financial limitations that restrict the performance of IPC – it appears some African hospitals are able to implement significant IPC activities within limited budgets. As others have also found ^19^, the range of IPCAF scores amongst these African hospitals was much wider than the range seen amongst much larger numbers of European hospitals in either Germany ^21^ or Austria ^22^. This suggests that across sub-Saharan Africa, hospital IPC activities are typically both less advanced and much more variable than within high-income nations.

The variation in the responses to the WHO stewardship toolkit were extreme with “yes” responses to the questions ranging from 2 to 23/31. The median score (14.5 “yes” responses) was less than 50% of the possible maximum score. This suggests antimicrobial stewardship activities in these eight hospitals were limited and highly variable. However, this tool was not designed with between-facility comparisons in mind but was rather intended to support national and facility-level development of stewardship activities through an iterative process of self-assessment. We have not found any published examples of multi-site studies using these questions from this WHO toolkit in this way.

The variation found in access to antibiotic agents through repeated cross-sectional surveys was substantial, ranging from universal access to all 11 drugs surveyed (in one hospital in South Africa) to intermittent access to the majority of these drugs (in the hospitals in Ghana, Malawi and Zambia) to a consistent-but-limited pattern of antibiotic access (in one hospital in Ethiopia). No two hospitals displayed the same pattern of results in these cross-sectional antibiotic access surveys, though the antibiotics ceftriaxone and ciprofloxacin were consistently available in all hospitals. We are not aware of any previous research that has investigated the availability of agents for treating invasive Enterobacterales infections in LMIC in this way. There was limited access to agents such as piperacillin-tazobactam, amikacin and meropenem that are all vital drugs for treating resistant Enterobacterales infections. As emphasized in a recent review, access to antimicrobials in LMIC hospitals is a serious issue ^23^. Intermittent drug availability in these tertiary hospitals is highly concerning as it suggests that health systems across the African continent are ill-equipped to treat severe antibiotic-resistant infections.

Apart from one hospital in South Africa, investigators at all hospitals described that patients were given prescriptions to buy antibiotics from private pharmacies outside the hospital using out-of-pocket expenditure. Additionally, for hospitals in Tanzania, Zambia and Ghana, patients could access some additional antibiotics from external private pharmacies for free if they were enrolled in the national insurance schemes. However, when comparing pharmacy drug availability against the actual use of antibiotics by patients with confirmed Enterobacterales BSI in these institutions, we found that there was a clear correlation between pharmacy availability and use of drug in individual patients. This suggests that in practice, external purchase of antibiotics for treating drug-resistant BSI took place relatively rarely in most of these hospitals during the study period. 

This study has several notable strengths through use of original approaches. This is, to the best of our knowledge, the first study to report use of the WHO stewardship toolkit from hospitals in multiple sub-Saharan African countries. We are not aware of any prior publication describing availability of antibiotic agents for treating infections in repeated pharmacy-based surveys in hospitals across multiple sub-Saharan African nations, nor of any prior work comparing pharmacy availability against patient-level use in this region. Infection control and access to antibiotics are both subjects of intense international interest as attention turns to how we can avert future deaths from bacterial AMR.

In terms of study limitations, firstly, these eight hospitals are unlikely to be representative of all hospitals in sub-Saharan Africa. These hospitals are mainly located in major urban areas with well-developed microbiology services and some participation in research studies. The hospitals in Kenya and Malawi both have long-standing relationships with international research organizations, unusual amongst African public-sector hospitals. This introduces a selection bias to our sample, which we believe is likely to give an overly-optimistic picture of services across the region. Secondly, while these data were collected by senior healthcare staff with research experience using standardized materials, we remain concerned about the possibility of response bias (in particular, a social-desirability bias) in these self-reported questionnaires, especially the WHO stewardship toolkit. This form of bias could lead to either over-optimistic or over-pessimistic responses, in comparison to an idealized external assessor. 

## Conclusion

Across eight tertiary hospitals in sub-Saharan Africa, we found wide variation in the intensive care facilities, the self-assessed IPC activities, stewardship activities and the objectively-assessed profile of access to antibiotic drugs for treating invasive Enterobacterales infections. This means that the same, or highly similar, antibiotic resistant pathogens could plausibly have very different human-level impacts between these hospitals. The ongoing MBIRA study is gathering patient-level outcome data from these same eight hospitals combined with individual-level antibiotic use data for treatment of Enterobacterales BSI. We hope that these and further data will provide the basis to better understand the human impacts of antibiotic resistance across sub-Saharan African nations.

## Consent for publication

This study is published with the permission of: the Director of KEMRI, Kenya; NIMR, Tanzania; and NHRA, Zambia

## Data Availability

LSHTM Data Compass: MBIRA - Mortality from Bacterial Infections Resistant to Antibiotics study - underlying data. https://doi.org/10.17037/DATA.00003168 ^24^ This project contains the following underlying data: mbira_hospital_data.xlsx (raw hospital data) mbira_pharmacy_V2.xlsx (raw pharmacy and collapsed patient data) Full participant is not able to be made publicly available under the terms of the LSHTM Ethics Committee’s agreement. Readers can apply for access via the link above, clicking ‘mbira_data’ and ‘request access’. Applicants will need to complete the request form including name, proposed use of data, data requested, supporting information, and email address. LSHTM Data Compass: MBIRA - Mortality from Bacterial Infections Resistant to Antibiotics study - underlying data. https://doi.org/10.17037/DATA.00003168 ^24^ This project contains the following extended data: Data-Codebook.html (data key for underlying data files) MBIRA_data_documentation_appendices.pdf (additional information including survey and consent forms) MBIRA_patient_information_child_English.pdf (consent form for parents of child participants) Data are available under the terms of the Creative Commons Attribution 4.0 International license (CC-BY 4.0). World Bank data is available from: https://data.worldbank.org/indicator/NY.GDP.PCAP.PP.CD Apply the filter ‘Current international US$’ and select the relevant year/country.
