# Supplementary material for: Circumstances for treatment and control of invasive Enterobacterales infections in eight hospitals across sub-Saharan Africa: a cross-sectional study
Source: Gates Open Res. 2023 Feb 1;7:21. [Version 1] doi: 10.12688/gatesopenres.14267.1 (PMC10963387; doi:10.12688/gatesopenres.14267.1)
Supplement: Peer review history

# Reviewer report: Reviewer response for version 1

**DOI:** 10.21956/gatesopenres.15578.r35944

**Published:** 2024-03-25

**Authors:** Nasreen Hassoun-Kheir

**Affiliations:** 1 Geneva University Hospitals and Faculty of Medicine, Geneva,, Switzerland

Thank you for the invitation to review this manuscript by Aiken et al; it provides important insights into the infection prevention and control practices, antimicrobial stewardship practice and availability of antibiotic agents across eight hospitals in sub-Saharan Africa. The evidence generated in this study is of great importance, considering the overall lack of empiric data on antimicrobial resistance in sub-Saharan Africa. The authors completed repeated cross-sectional hospital assessments of practice and availability of antibiotics and compared the findings with individual-patients antibiotic consumption in patients with Enterobacterales bloodstream infection. The manuscript is clear and well structured, yet there are some minor comments that need to be clarified:

- The study setting is of particular interest, were all hospital approached to participate in the study tertiary care centers? How were they identified?
- As data on antibiotic treatments was derived from patients who gave consent to participate in the study, it is important to report on what was the overall proportion of the enrolled/eligible population? Did it differ between centers?
- According to the study results major area of weakness was identified in ‘hospital-acquired infections surveillance’ and ‘workload, staffing and bed occupancy’ components. This was also observed in another study in the area. Can the authors provide more details about this? for example in CC4 Health care-associated infection surveillance, was the problem lack of personnel? Training? Resources?
- How were blood culture costs managed during the study? Did patients pay for the tests? Were they funded by the study/medical insurance?
- As the study was conducted during the COVID pandemic, do the authors think that this might have affected their findings?
- Figure 1 depicting pharmacy antibiotics availability and antibiotic treatments is crowded and hard to understand, it would be better to display the correlation per antibiotic agent/class separately in multiple figures. 

Is the work clearly and accurately presented and does it cite the current literature?

Yes

If applicable, is the statistical analysis and its interpretation appropriate?

Yes

Are all the source data underlying the results available to ensure full reproducibility?

Yes

Is the study design appropriate and is the work technically sound?

Yes

Are the conclusions drawn adequately supported by the results?

Yes

Are sufficient details of methods and analysis provided to allow replication by others?

Yes

Reviewer Expertise:

Infectious diseases, epidemiology, infection prevention and control, antimicrobial resistance

I confirm that I have read this submission and believe that I have an appropriate level of expertise to confirm that it is of an acceptable scientific standard.

# Reviewer report: Reviewer response for version 1

**DOI:** 10.21956/gatesopenres.15578.r32905

**Published:** 2023-08-10

**Authors:** Catrin E. Moore

**Affiliations:** 1 Centre for Neonatal and Paediatric Infection, St. George’s University of London, London, UK

This publication on the MBIRA study describes different hospital management of blood stream infections caused by resistant Enterobacterales in eight public sector hospitals across sub-Saharan Africa. All of the hospitals were enrolled in the MBIRA research study, and this baseline assessment examined the antibiotic availability and use within the hospital pharmacies, together with prescriptions and use by patients. The study found a heterogeneity in the management of resistant blood stream infections, with differences in the infection prevention level and antibiotic stewardship activities which didn’t correlate with national income levels. There was a variation in the available antibiotics, however the use of the antibiotics correlated well with their availability in the hospital pharmacy, suggesting in this particular scenario within these hospitals that antibiotics were not purchased outside the hospital.

 The publication is well written, I only have a few minor comments.

**Methods; Data collection:**

 You mention different scores in Ghana, but how different were the scores of the 7 specialists who were recording the WHO-ICPAF survey in Ghana?

**Results:**

 It would be useful to have start and end days as well as months and years for the hospital data collection.

**Figure 1:** scatter plot, although the idea is good in principle, in practice it’s difficult to see the different antibiotic classes and countries, a single letter or different shapes for the antibiotic class would be easier to understand

 You currently have a reference for figure 2 earlier in the text than figure 1, the numbering needs to be updated so figure 1 is first.

Is the work clearly and accurately presented and does it cite the current literature?

Yes

If applicable, is the statistical analysis and its interpretation appropriate?

Yes

Are all the source data underlying the results available to ensure full reproducibility?

Yes

Is the study design appropriate and is the work technically sound?

Yes

Are the conclusions drawn adequately supported by the results?

Yes

Are sufficient details of methods and analysis provided to allow replication by others?

Yes

Reviewer Expertise:

Infectious disease epidemiology, microbiology, global health

I confirm that I have read this submission and believe that I have an appropriate level of expertise to confirm that it is of an acceptable scientific standard.
